# Complexation and bonding studies on [Ru(NO)(H_2_O)_5_]^3+^ with nitrate ions by using density functional theory calculation†

**DOI:** 10.1039/d0ra05042c

**Published:** 2020-06-26

**Authors:** Akane Kato, Masashi Kaneko, Satoru Nakashima

**Affiliations:** Graduate School of Advanced Science and Engineering, Hiroshima University 1-3-1, Kagamiyama Higashi-Hiroshima Hiroshima 739-8526 Japan; Nuclear Science and Engineering Center, Japan Atomic Energy Agency 2-4, Shirakata, Tokai-mura Ibaraki 319-1195 Japan kaneko.masashi@jaea.go.jp; Natural Science Center for Basic Research and Development, Hiroshima University 1-4-2, Kagamiyama Higashi-Hiroshima Hiroshima 739-8526 Japan snaka@hiroshima-u.ac.jp

## Abstract

Complexation reactions of ruthenium–nitrosyl complexes in HNO_3_ solution were investigated by density functional theory (DFT) calculations in order to predict the stability of Ru species in high-level radioactive liquid waste (HLLW) solution. The equilibrium structure of [Ru(NO)(NO_3_)_3_(H_2_O)_2_] obtained by DFT calculations reproduced the experimental Ru–ligand bond lengths and IR frequencies reported previously. Comparison of the Gibbs energies among the geometrical isomers for [Ru(NO)(NO_3_)_*x*_(H_2_O)_5−*x*_]^(3−*x*)+/−^ revealed that the complexation reactions of the ruthenium–nitrosyl complexes with NO_3_^−^ proceed *via* the NO_3_^−^ coordination to the equatorial plane toward the Ru–NO axis. We also estimated Gibbs energy differences on the stepwise complexation reactions to succeed in reproducing the fraction of Ru–NO species in 6 M HNO_3_ solution, such as in HLLW, by considering the association energy between the Ru–NO species and the substituting ligands. Electron density analyses of the complexes indicated that the strength of the Ru–ligand coordination bonds depends on the stability of the Ru species and the Ru complex without NO_3_^−^ at the axial position is more stable than that with NO_3_^−^, which might be attributed to the difference in the *trans* influence between H_2_O and NO_3_^−^. Finally, we demonstrated the complexation kinetics in the reactions *x* = 1 → *x* = 2. The present study is expected to enable us to model the precise complexation reactions of platinum-group metals in HNO_3_ solution.

## Abstract 2

Density functional study on the complexation of [Ru(NO)(H_2_O)_5_]^3+^ with NO_3_^−^ ions reproduced the stabilities of the geometrical isomers and the stepwise substitution reactivities by combining the association energy with the leaving/entering ligands.

## Graphical abstract

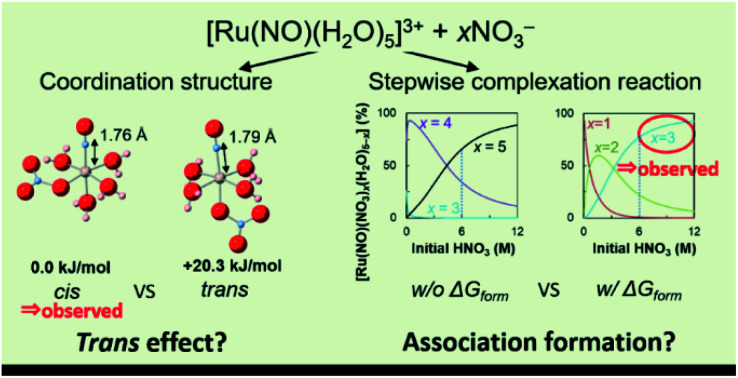

## Introduction

High-level liquid waste (HLLW) is an aqueous solution of 2–6 M HNO_3_ including various kinds of metal ions, generated during reprocessing of spent nuclear fuel. Ruthenium (Ru), rhodium (Rh), and palladium (Pd), called platinum-group metals (PGMs), are included in the HLLW solution and have been known to precipitate with coexisting ions, such as molybdenum and zirconium.^1^ This is considered to hinder the vitrification process of HLLW^2^ as well as continuous solvent extraction for the partitioning and transmutation strategy of HLLW.^3^ PGMs have also structural diversity and redox properties to work as the catalysts for organic syntheses^4^ and selective reduction of NO_*x*_.^5^ The catalytic activity has been indicated to reduce the hydrogen-generation in HLLW solution as well as to generate NO_2_ and NO.^6^ The efficient separation of PGMs from HLLW has been studied for the efficient disposal of HLLW and reuse as the PGM resource by means of solvent extraction, ionic exchange, and the other techniques.^7–9^ In order to understand the separation mechanism, the coordination species of PGMs in HLLW solution need to be elucidated.

PGM ions form the complexes with H_2_O and NO_3_^−^ ligands in nitric acid solution. The composition of the coordination species varies depending on the concentration of nitric acid and the reaction time and at the most three or four equivalents of NO_3_^−^ coordinates to one equivalent of PGM ion in concentrated nitric acid solution.^10–15^ Focusing on the case of Ru system, the Ru exists as trivalent ruthenium–nitrosyl ions, [Ru(NO)]^3+^, which consists of divalent Ru ion (Ru^II^) and NO^+^ ion, in nitric acid solution and forms six-coordinated octahedral complexes, resulting in [Ru(NO)(NO_3_)_*x*_(H_2_O)_*y*_]^(3−*x*)+/−^ (*x* + *y* = 5 for unidentate NO_3_ coordination). When considering the complexes with *x* = 0–4, the number of geometrical isomers increases to 11 for the unidentate system. Variation of the isomers becomes enormous in HLLW solution and this makes it difficult to understand systematically the structural parameters and reactivity of the ruthenium–nitrosyl complexes as well as those of Rh and Pd systems, because nitrite ion, NO_2_^−^, also exists in HLLW and works as a ligand possessing both nitro-*N* (nitrogen-donor) and nitrito-*O* (oxygen-donor) coordination styles. However, such systematic properties of the ruthenium–nitrosyl complexes, even if limited to [Ru(NO)(NO_3_)_*x*_(H_2_O)_*y*_]^(3−*x*)+/−^ system, has not been understood, although the precise modeling of coordination geometries in nitric acid solution is required to understand the separation mechanism in solvent extraction.^16^

Density functional theory (DFT) calculations have been employed to interpret experimental results and to understand the coordination character of PGM complexes.^17,18^ Recently, the DFT applications to the Rh and Pd complexes with H_2_O and NO_3_^−^ ligands, considering the geometrical isomers for the unidentate system, have been reported by combining them with experimental techniques.^19,20^ Watanabe *et al.* studied the coordination bond lengths and electronic transitions of [Pd(NO_3_)_*x*_(H_2_O)_4−*x*_]^(2−*x*)+/−^ by combining EXAFS and UV-Vis spectroscopies with DFT and first-principles many-electrons calculations.^19^ Vasilchenko *et al.* investigated the complexation reactions of [Rh(NO_3_)_*x*_(H_2_O)_6−*x*_]^(3−*x*)+/−^ and [Pd(NO_3_)_*x*_(H_2_O)_4−*x*_]^(2−*x*)+/−^ by combining ^15^N-NMR spectroscopy with DFT calculations.^20^ We also have published a DFT benchmark study on ruthenium–nitrosyl complexes, [Ru(NO)L_5_] (L = Br^−^, Cl^−^, NH_3_, and CN^−^), with previously reported ^99^Ru Mössbauer spectroscopic parameters and correlated the Mössbauer isomer shift and quadrupole splitting values with the ligand field strength by DFT-based electron density analyses.^21^

Present study applies the DFT method optimized by the previous benchmark study to the ruthenium–nitrosyl complexes with H_2_O and NO_3_^−^ ligands for modeling the stable coordination structures and complexation reactions of NO_3_^−^ ligands toward [Ru(NO)(H_2_O)_5_]^3+^ when assuming strong nitric acid solution, such as HLLW solution (but not considering coordination of NO_2_^−^ and OH^−^ ligands as well as optical isomers for simplicity). We also discuss origins of stability in the structures between geometrical isomers and in the stepwise formation reactions with NO_3_^−^ by using Gibbs energy and coordination bonding analyses.

## Computational details

**Fig. 1:**
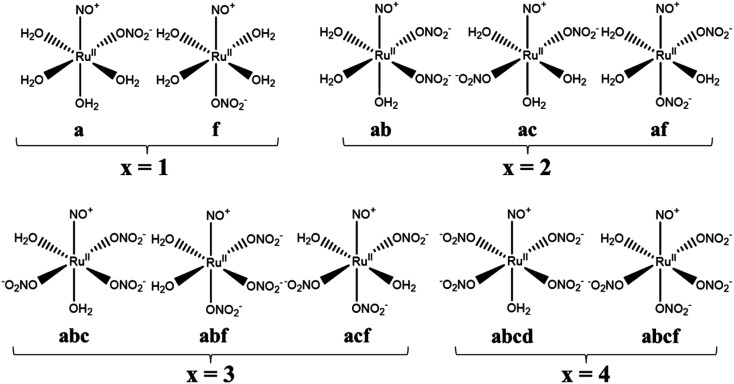
Structural formulas and abbreviations of geometrical isomers of [Ru(NO)(NO_3_)_*x*_(H_2_O)_5−*x*_]^(3−*x*)+/−^.

Calculation model complexes, [Ru(NO)(NO_3_)_*x*_(H_2_O)_5−*x*_]^(3−*x*)+/−^ (*x* = 0–5), were based on single crystal X-ray coordinates with the analogous coordination geometries obtained from Cambridge Structural Database (CSD).^22^ The complex with *x* = 0 was modeled by using the coordination sphere of [Ru(CO)(H_2_O)_5_](CH_3_C_6_H_4_SO_3_)_2_ (CSD code: EBIDAU^23^) and the complexes with *x* = 1–5 were modeled by using those of [Pt(NO_3_)_6_](C_5_H_5_NH)_2_ (CSD code: CIMVEB^24^). The starting structures were created by cutting off only octahedral coordination sphere from the crystal coordinates by using VESTA program ver. 4.3.0,^25^ followed by replacing the corresponding functional groups by using Winmostar program ver. 9.4.0.^26^ Geometrical isomers of [Ru(NO)(NO_3_)_*x*_(H_2_O)_5−*x*_]^(3−*x*)+/−^ exist in cases of the complexes with *x* = 1–4. All the abbreviations were named by defining the nitrosyl position as “*e*” and the orthogonal plane as “*abcd*” based on IUPAC nomenclature,^27^ summarized in Fig. 1. In case of the complex with *x* = 1, there are two isomers whether one NO_3_^−^ coordinates to “*a*” or “*f*” position, defined as a and f, respectively. Three isomers exist for the complexes with *x* = 2 and 3 and named as ab, ac, af for *x* = 2 system, and abc, abf, acf for *x* = 3 system. The complex with *x* = 4 has two isomers, abcd and abcf. All the Cartesian coordinates of the optimized structures are available in ESI.†

All DFT calculations were performed by the ORCA program ver. 3.0.^28^ The electronic ground state of the ruthenium–nitrosyl complexes was considered as low-spin state with Ru^2+^(4d^6^)–NO^+^ configuration, *i.e.* singlet state (t^6^_2g_), because our pervious DFT results for several ruthenium–nitrosyl complexes, [Ru(NO)L_5_] (L = Br^−^, Cl^−^, NH_3_, and CN^−^), of the singlet state reproduced the experimental data, such as the metal–ligand bond lengths, bond angles, IR frequencies, and ^99^Ru Mössbauer spectroscopic parameters.^21^ The DFT methods for geometry optimizations and single-point energy calculations and the electron density analyses were carried out based on the condition previously reported.^21^ Segmented all-electron relativistically contracted (SARC) Gaussian-type basis set^29^ optimized for the corresponding relativistic correction was assigned to all atoms to perform all-electron scalar-relativistic DFT calculations. SARC basis set was used as the recontracted version implemented in ORCA: the exponents are cited in ref. 30 and the recontraction method can be obtained in ref. 29. SARC basis set of Ru atom was assigned as (17s13p8d) contracted to (12s9p4d) for geometry optimization, and (19s15p9d) contracted to (12s10p5d) for single point energy calculation. In geometry optimization and vibrational frequency modes calculations, we employed the split valence plus one polarization (SVP) basis set^29^ for O, N, C, and H atoms with Becke88 exchange^31^ and Perdew86 correlation^32^ functional (BP86) at zeroth-order regular approximation (ZORA) level.^33,34^ We checked that the optimized models were converged to be at local minimum geometries, *i.e.*, the models have no imaginary vibrational modes. In single-point energy calculations and the electron density analyses, triple-zeta valence plus one polarization (TZVP) basis sets^30^ were assigned to O, N, C, and H atoms with B3LYP functional^35^ in a framework of second-order Douglas–Kroll–Hess (DKH2) Hamiltonian^36^ by using the equilibrium structures. The conductor-like screening model (COSMO)^37^ of water solvent (relative permittivity: 80.4, refractive index: 1.33) was applied for all the DFT calculations in order to consider a dielectric polarizable continuum of solvent for both geometry optimization and single-point energy calculations. The resolution of the identity (RI) approximation was employed for pure DFT and hybrid DFT calculations as Split-RI-J^38^ and RIJCOSX^39^ methods, respectively.

The Gibbs energies were analyzed by using previously published method.^40^ All the thermal correction terms in the Gibbs energies were based on the thermochemical concept of molecular partition functions,^41^ assuming the standard condition (298.15 K, 1.0 atom). The vibrational and rotational contributions to the thermal correction were obtained by harmonic oscillator and rigid rotator approximations, respectively. We considered the quasi-harmonic approximation of the vibrational contribution by unifying the normal vibrational frequencies lower than 60 cm^−1^, because this method is known to correct the well-known breakdown of the harmonic oscillator model for the free energies of low-frequency vibrational modes.^42,43^ The computational details of the thermal correction are mentioned in ESI.† Bond critical point (BCP) analyses based on “quantum theory in atoms in molecules” (QTAIM)^44^ were performed for understanding the strength of the Ru–ligand coordination bonds by using NBO program ver. 6.0.^45^ The analyses of natural population^46^ and density of states (DOS)^47^ were also carried out in order to evaluate the bonding strength. Three-dimensional (3D) descriptions of the optimized structures and MO surfaces were illustrated by the VESTA program ver. 4.3.0.^25^

## Results and discussion

### Equilibrium structures of [Ru(NO)(NO_3_)_*x*_(H_2_O)_5−*x*_]^(3−*x*)+/−^

Ru–ligand bond lengths of [Ru(NO)(NO_3_)_*x*_(H_2_O)_5−*x*_]^(3−*x*)+/−^ (*x* = 0–5) are summarized in Table 1. Comparing the Ru–N(NO) and Ru–O_all_ bond lengths among all the complexes, the values were 1.76–1.78 Å and 2.07–2.08 Å, respectively, and the complexes were indicated to have similar coordination bonds each other. Experimental bond lengths of the complex with *x* = 3, which was fitted as the abc model, in solution were reported by using EXAFS measurement as 2.04(3), 2.11(9), and 2.08(1) Å for Ru–O_eq_(NO_3_), Ru–O_eq_(H_2_O), and Ru–O_ax_(H_2_O) bond lengths, respectively.^16^ The corresponding calculated values, 2.06(3), 2.13, and 2.08 Å, respectively, were consistent with the experimental values within the standard deviation. We also showed *Σ* values, which denote the sum of the deviations from the ideal octahedron of the *cis* Ru–ligand bond angles in Table 1. Comparing the *Σ* values among the geometrical isomers, the complex with coordination of NO_3_^−^ to the equatorial position have greater *Σ* value than that to the axial position. Furthermore, the increase of replacement of NO_3_^−^ from H_2_O into equatorial position was indicated to make the *Σ* values larger. All the bond lengths and angles are summarized in Table S1.†

**Table 1:** Calculated Ru–ligand bond lengths of [Ru(NO)(NO_3_)_*x*_(H_2_O)_5−*x*_]^(3−*x*)+/−^

Complexes		Bond lengths^a^/Å				*Σ* b/deg.
		Ru–N(NO)	Ru–O(NO_3_)	Ru–O(H_2_O)	Ru–O_all_	
*x* = 0		1.768	—	2.065(14)	2.065(14)	54.4
*x* = 1	a	1.762	2.012	2.087(26)	2.072(38)	59.4
	f	1.785	2.006	2.92(2)	2.075(34)	41.4
*x* = 2	ab	1.765	2.033	2.107(20)	2.077(42)	61.5
	ac	1.761	2.064	2.089(8)	2.079(18)	61.3
	af	1.772	2.029	2.104(19)	2.074(40)	47.9
*x* = 3	abc	1.760	2.058(25)	2.108	2.078(37)	71.4
	abf	1.765	2.041(12)	2.130	2.076(44)	67.4
	acf	1.764	2.059(17)	2.095	2.076(22)	53.5
*x* = 4	abcd	1.756	2.073(28)	2.096	2.077(26)	80.0
	abcf	1.759	2.062(16)	2.139	2.077(34)	66.8
*x* = 5		1.761	2.074(16)	—	2.074(16)	78.7

a Parentheses show the standard deviation to the averaged value.

b Sum of deviations of the *cis*-L–Ru–L bond angles from 90 deg.

We estimated the IR vibrational frequencies of the complexes with *x* = 3 as averaged sum weighted by calculated IR intensities by analyzing the normal vibrational frequency modes. Table 2 shows the calculated IR frequencies and assignment of normal vibrational modes with the experimental values in solid state.^12,16^ The calculated frequencies of three isomers show the similar values to the experimental frequencies, especially, those of the abc model were found to well reproduce the O–H stretching vibration compared to the other isomers. The IR frequencies and intensities of normal vibrational modes employed for the estimation are summarized in Table S2.†

**Table 2:** Comparison of IR frequencies of the complexes with *x* = 3 between calculation and experiment

Calc./cm^−1^			Exp./cm^−1^	Assignment
abc	abf	acf		
743	739	729	765^a^	*δ*_s*y*m_(NO_3_), Ru–H_2_O rocking
770	769	763	783^a^	*γ*_s*y*m_(NO_3_)
915	916	912	968^a^, 963^b^	*δ*_s*y*m_(NO_3_)
1251	1272	1252	1265^a^, 1268^b^	*δ*_s*y*m_(NO_3_), *ν*_s*y*m_(NO_3_)
1523	1555	1534	1508^a^, 1527^b^	*γ*_s*y*m_(H_2_O)
1615	1609	1612	1620^a^, 1670^a^	*ν*_as*y*m_(NO_3_)
1967	1954	1962	1945^a^, 1932^b^	*ν*(NO)
3061	2983	2946	3140^a^	*ν*(H_2_O)

a Reference 12.

b Reference 16.

### Gibbs energy analyses

Table 3 shows the relative Gibbs energies, *G*^rel^_*x*_, in which the most stable isomer is set to 0.0 kJ mol^−1^, with the Boltzmann distributed fraction based on the *G*^rel^_*x*_ values for the geometrical isomers of [Ru(NO)(NO_3_)_*x*_(H_2_O)_5−*x*_]^(3−*x*)+/−^ (*x* = 1–4). When comparing the *G*^rel^_*x*_ values among the isomers, the complexes possessing the NO_3_ ligands at the equatorial (*a*, *b*, *c*, *d*) position, *i.e.* the complexes a, ab or ac, abc, abcd for *x* = 1, 2, 3, 4, respectively, are the most stable than those having the axial (*f*) NO_3_ ligands and the fraction is greater than 70%. This supports the identification of these isomers by Scargill *et al.*^12^ In case of the complexes with *x* = 2, the ab isomer was found to be more stable than the ac isomer, being consistent with the previous study, though the Boltzmann distributed fraction of the complex ab compared to the complex ac, which is 5.3, did not match with the observed fraction, 1.4.^12^ In case of the complexes with *x* = 3, this result supports that the estimated Ru–ligand bond lengths were fitted as the abc model by EXAFS measurement^16^ and the calculated O–H stretching frequency of the abc model was closer to the experimental values^12,16^ compared to the other isomers shown in Table 2. It is also interesting that the fraction of the most stable isomers decreases as the NO_3_ coordination proceeds, the complexes a (100%) → ab (80%) → abc (76%) → abcd (70%). We considered that the distortion of the Ru octahedron caused by NO_3_ coordination into equatorial plane observed in the increase of the *Σ* value (Table 1) originates in the relative destabilization of the most stable isomers, resulting in the competed *G*_rel_ values among the isomers. All the numerical values of thermodynamic parameters are summarized in Table S3.†

**Table 3:** Calculated values of *G*^rel^_*x*_, ΔG^overall^_*x*_, and Δ*G*^stepwise^_*x*_ under standard condition

Complexes		*G* ^rel^_*x*_/kJ mol^−1^^a^	ΔG^overall^_*x*_/kJ mol^−1^	Δ*G*^stepwise^_*x*_/kJ mol^−1^^b^	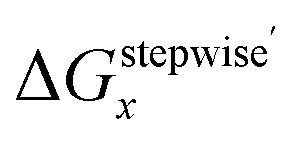 /kJ mol^−1^^c^
*x* = 1	a	0.0 (100)	−84.6	−84.6	−29.5 (*x* = 1 → a)
	f	20.3 (0)	−64.3		
*x* = 2	ab	0.0 (80)	−143.3	−55.8	−13.5 (a → ab)
	ac	4.1 (15)	−139.2		
	af	7.0 (5)	−136.3		
*x* = 3	abc	0.0 (76)	−192.5	−49.1	−6.4 (ab → abc)
	abf	7.2 (4)	−185.3		
	acf	3.3 (20)	−189.2		
*x* = 4	abcd	0.0 (70)	−213.3	−21.1	11.6 (abc → abcd)
	abcf	2.1 (30)	−211.2		
*x* = 5		—	−218.3	−5.7	25.1 (abcd → *x* = 5)

a Parenthese show Boltzmann distribution fraction (%) based on the *G*_rel_ values among the isomers.

b Estimated based on Boltzmann averaged Gibbs energies.

c Values based on Gibbs energy differences for the reaction paths shown in parentheses.

We analyzed the Gibbs energy differences in the complex formation reaction of [Ru(NO)(H_2_O)_5_]^3+^ with NO_3_^−^ ligands. The Gibbs energy differences are defined as Δ*G*^overall^_*x*_ and Δ*G*^stepwise^_*x*_ for the overall and stepwise formation reaction, respectively, described as eqn (1) and (2), respectively. The Δ*G*^stepwise^_*x*_ values were estimated by using the Boltzmann averaged Gibbs energies based on relative Gibbs energies among the isomers and shown in Table 3. The values are found to be negative for the cases of *x* = 1–5, indicating that the fifth NO_3_ complexation reaction proceeds spontaneously. This tendency of Gibbs energy differences was consistent with that of stepwise complexation of NO_3_^−^ toward [Pd(H_2_O)_4_]^2+^ estimated by DFT calculation.^19^ The corresponding Δ*G*^stepwise^_*x*_ values were reported as −98.8 (*x* = 0 → *x* = 1), −48.6 (*x* = 1 → *trans*), −50.3 (*trans* → *x* = 3), −18.9 kJ mol^−1^ (*x* = 3 → *x* = 4). The absolute values were, however, greater than 6 times as those of the corresponding experimental values, −10.1, −7.1, −6.0, −1.7 kJ mol^−1^, respectively, based on the overall formation constants.^20^

1 [Ru(NO)(H_2_O)_5_]^3+^ + *x*NO_3_^−^ ⇌ [Ru(NO)(NO_3_)_*x*_(H_2_O)_5−*x*_]^(3−*x*)+/−^ + *x*H_2_O

2 [Ru(NO)(NO_3_)_*x*−1_(H_2_O)_6−*x*_]^(4−*x*)+/−^ + NO_3_^−^⇌ [Ru(NO)(NO_3_)_*x*_(H_2_O)_5−*x*_]^(3−*x*)+/−^ + H_2_O

Mismatching of the Δ*G*^stepwise^_*x*_ might attribute to an overstabilization of the coordination species of the final state after the exchange reaction from H_2_O to NO_3_^−^ because of the lowering the charge of the complexes. In order to improve the overstabilization and compensate the difference in the charge of the complexes during the exchange reaction, we introduced formation energies of an association, A⋯B, between a coordination species (A) and a molecule (B), which is exchanged in the complexation reaction, *i.e.* the association formation between [Ru(NO)(NO_3_)_*x*−1_(H_2_O)_6−*x*_]^(4−*x*)+/−^ and NO_3_^−^ into initial state and that between [Ru(NO)(NO_3_)_*x*_(H_2_O)_5−*x*_]^(3−*x*)+/−^ and H_2_O into final state for the stepwise formation. For simplicity we considered the energies for the stepwise complexation reactions through the most stable isomers among the composition shown in Tables 3, *i.e.* the complexes *x* = 0 → a → ab → abc → abcd → *x* = 5, because the stepwise reaction path is considered to proceed advantageously, which was supported by experiment.^12^ The position of the molecule B was determined by using unconstrainted geometry optimization with relaxed surface scanning. We estimated the formation energy, *G*_form_ (A⋯B), as the Gibbs energy difference described in eqn (3). The corrected Δ*G*^stepwise^_*x*_ values, 
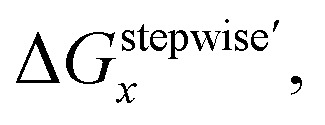
, in which the Δ*G*_form_ values were added to Δ*G*^stepwise^_*x*_, were calculated by using eqn (4) and (5) and shown in Table 3. The values were positive when *x* = 4 and 5, unlike the result of the Δ*G*^stepwise^_*x*_, indicating that the complex formation reaction is hard to proceed after the third NO_3_ coordination. This result explains that the complex with *x* = 3 exists as major component even the fraction is higher than 50% in concentrated HNO_3_ solution (>10 M).^11,12,48^ The introduction of *G*_form_(A⋯B) makes the Δ*G*^stepwise^_*x*_ values more positive and the Δ*G*_form_(A⋯B) values are larger for the smaller *x*. This might be due to the relative stabilization of initial states during the Δ*G*^stepwise^_*x*_ for *x* = 1 and 2 by considering the interaction between a cationic Ru species and NO_3_^−^. The numerical data of *G*_form_(A⋯B) and Δ*G*_form_(A⋯B) are shown in Table S4.†

3 *G*_form_(A⋯B) = *G*(A⋯B) − {*G*(A) + *G*(B)}

4 Δ*G*_form_(A⋯B) = *G*_form_(A⋯B)_final_ − *G*_form_(A⋯B)_initial_

5
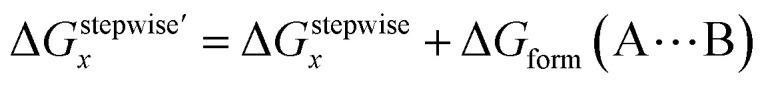


### Fractions of Ru species based on Δ*G*^stepwise^_*x*_

Based on the stepwise complexation Gibbs energy differences, Δ*G*^stepwise^_*x*_ and 
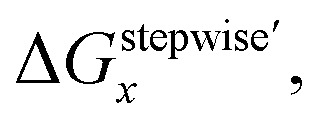
 we demonstrate to simulate the dependency of the fractions of the [Ru(NO)(NO_3_)_*x*_(H_2_O)_5−*x*_]^(3−*x*)+/−^ species on initial HNO_3_ concentration by using the following eqn (6)–(11). We assumed the activity coefficients of the Ru species as 1 to give the stepwise complex formation constant, *K*_*x*_ (*x* = 1–5), in eqn (7), in which *a*_A_ and *C*_A_ denote the activity and concentration of molecule A at equilibrium, respectively, because the total Ru concentration (*C*^tot^_Ru_) in HLLW solution is low (*ca.* ∼10^−2^ M). We also approximated that the *C*^tot^_Ru_ is lower than the total concentrations of HNO_3_ (*C*_HNO_3__^tot^) and H_2_O (*C*_H_2__^tot^_O_) enough to be ignored to obtain eqn (8) and (9) under such the experimental conditions previously reported^11,12,48^ as well as in HLLW solution. By using above assumptions the concentrations of [Ru(NO)(H_2_O)_5_]^3+^ and [Ru(NO)(NO_3_)_*x*_(H_2_O)_5−*x*_]^(3−*x*)+/−^ (*x* = 1–5) at equilibrium are described as eqn (10) and (11), respectively. We also compared two fitting methods to describe the activities of H_2_O and NO_3_^−^ (i) by using the activities based on the experimentally reported values^49^ (Method 1); (ii) by using the activities assuming the activity constants as 1 for simplicity (Method 2). We checked that the both fitting procedures are valid to fit with the dependency of the experimental fractions of [Ru(NO)(NO_3_)_*x*_(H_2_O)_5−*x*_]^(3−*x*)+/−^ (*x* = 0–4) on total HNO_3_ concentration by Scargill and coworkers.^12^ The fitting results and details are mentioned in ESI.†

6 Δ*G*^stepwise^_*x*_ = −*RT* ln *K*_*x*_

7
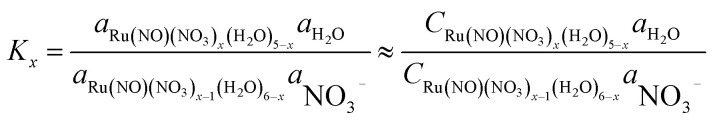
8
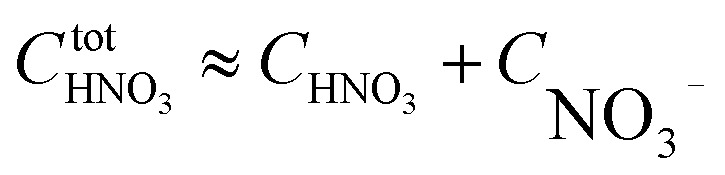
9
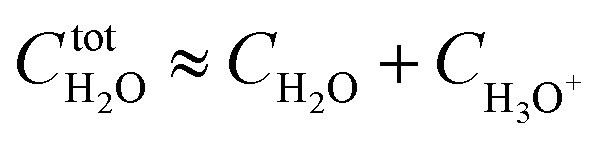
10
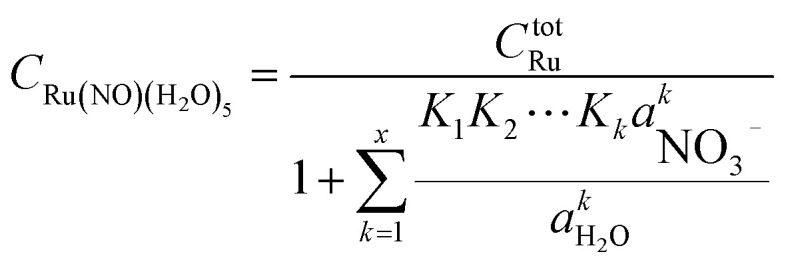
11
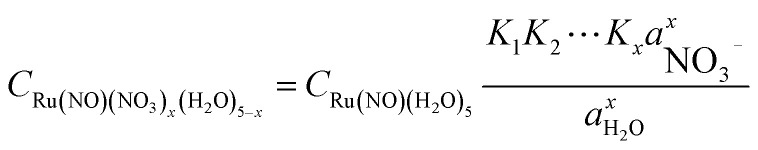


**Fig. 2:**
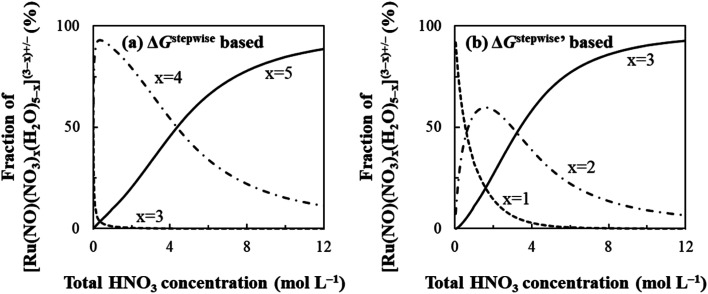
Dependency of fractions of [Ru(NO)(NO_3_)_*x*_(H_2_O)_5−*x*_]^(3−*x*)+/−^ on initial HNO_3_ concentration based on (a) Δ*G*_*x*_^stepwise^ and (b) 
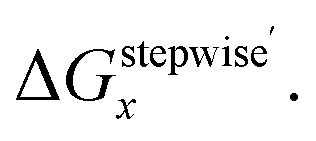

The simulation results of the Ru species fractions based on the Δ*G*^stepwise^_*x*_ and 
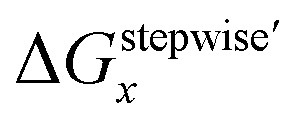
 are shown in Fig. 2a and b, respectively, by using the fitting Method 1. We found that the major products under the condition of higher than 2 M HNO_3_ were the complexes with *x* = 4 and *x* = 5 for the Δ*G*^stepwise^_*x*_ case, whereas *x* = 2 and *x* = 3 for the 
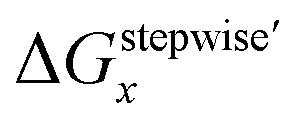
 case. Especially, the 
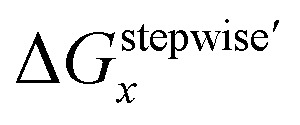
 based result reproduced that the complex with *x* = 3 exists as the most common component under the condition of higher than 6 M HNO_3_.^11,12,47^ We also compared the calculated fractions of the complexes under the condition of 6 M HNO_3_, such as in HLLW, with the experiments in Table 4. This indicates that the calculated fractions based on the corrected 
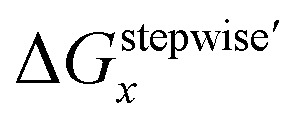
 reproduced the major components observed experimentally, *x* = 2 and *x* = 3, compared to those on the Δ*G*^stepwise^_*x*_ for the both fitting models. The present study implies that the model introducing the association formation energy improves the overstabilization based on the difference in the charge of the complexes, resulting in a valid estimation of the fractions of the coordination species for complex formation reaction.

**Table 4:** Fractions of [Ru(NO)(NO_3_)_*x*_(H_2_O)_5−*x*_]^(3−*x*)+/−^ under 6 M HNO_3_ condition under standard condition

Model		Fractions of [Ru(NO)(NO_3_)_*x*_(H_2_O)_5−*x*_]^(3−*x*)+/−^ (%)					
		*x* = 0	*x* = 1	*x* = 2	*x* = 3	*x* = 4	*x* = 5
Calc. 1^a^	Method 1	0.0	0.0	0.0	0.0	34.0	65.9
	Method 2	0.0	0.0	0.0	0.1	45.6	54.3
Calc. 2^b^	Method 1	0.0	0.7	22.2	76.9	0.2	0.0
	Method 2	0.0	0.8	31.7	67.4	0.1	0.0
Exp. 1^c^		3.7	30.0	24.0	41.7	—	—
Exp. 2^d^		1.0	9.0	46.0	38.5	5.5	—

a Values based on Δ*G*_*x*_^stepwise^ values.

b Values based on 
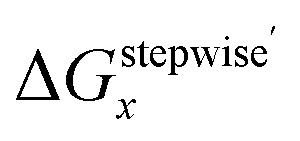
 values.

c Values of 5.96 M HNO_3_ at 293.15 K in ref. 11.

d Values of 6 M HNO_3_ at 293.15 K in ref. 12.

### Bonding analyses of [Ru(NO)(NO_3_)_*x*_(H_2_O)_5−*x*_]^(3−*x*)+/−^

We analyzed the atomic charge and electron configuration of Ru in the complexes [Ru(NO)(NO_3_)_*x*_(H_2_O)_5−*x*_]^(3−*x*)+/−^, shown in Table 5. The natural atomic charge, *ρ*_atom_(Ru), slightly decreased from *x* = 0 to *x* = 3 and unchanged from *x* = 3 to *x* = 5. This variation was caused by electron donation of the NO_3_^−^ ligands to the valence orbitals of Ru atom, mainly the d-orbitals, as shown in the variation of the orbital contribution to Ru electron configuration in Table 5. We also showed electron densities at BCP, *ρ*_BCP_, for the Ru–ligand bonds in Table 5. When comparing the total *ρ*_BCP_ values among the geometrical isomers, the complex with the largest value was a, ab, abc, and abcd for the complexes with *x* = 1, 2, 3, and 4, respectively. These complexes were indicated to be the most stable isomers by Gibbs energy analysis shown in Table 3. This also supports the experimental observation that NO_3_^−^ ligands are hard to coordinate to the *trans* position of the NO^+^ ligand.^12^ Especially, the total *ρ*_BCP_ value of the complex a was observed to be significantly greater than that of the complex f, caused by the difference in the *ρ*_BCP_ of Ru–NO bond. This is considered to lead to the shorter Ru–NO bond length of the complex a (1.762 Å) than that of the complex f (1.785 Å) shown in Table 1. This suggests that the stability among the geometrical isomers depends on the strength of the Ru–ligands bonds, especially the Ru–NO bond.

**Table 5:** Natural atomic charge, *ρ*_atom_, and orbital contribution to electron configuration for Ru atom, and electron density at BCP, *ρ*_BCP_, for Ru–ligand bonds of [Ru(NO)(NO_3_)_*x*_(H_2_O)_5−*x*_]^(3−*x*)+/−^

Complexes		*ρ*_atom_(Ru)/e	Orbital contribution/e			*ρ*_BCP_(Ru–ligand)/e bohr^−3^			
			s	p	d	Total	Ru–N(NO)	Ru–O(NO_3_)^total^	Ru–O(H_2_O)^total^
*x* = 0		1.012	0.265	0.009	6.714	0.6162	0.1748	—	0.4414
*x* = 1	a	0.985	0.273	0.011	6.731	0.6232	0.1782	0.1064	0.3386
	f	0.979	0.270	0.010	6.741	0.6080	0.1674	0.1052	0.3354
*x* = 2	ab	0.967	0.278	0.011	6.743	0.6224	0.1772	0.2014	0.2438
	ac	0.962	0.282	0.012	6.744	0.6202	0.1788	0.1856	0.2558
	af	0.962	0.278	0.012	6.748	0.6212	0.1744	0.1982	0.2486
*x* = 3	abc	0.952	0.287	0.013	6.749	0.6264	0.1798	0.2814	0.1652
	abf	0.960	0.284	0.012	6.745	0.6256	0.1782	0.2924	0.1550
	acf	0.956	0.282	0.013	6.749	0.6256	0.1786	0.2766	0.1704
*x* = 4	abcd	0.958	0.290	0.013	6.740	0.6292	0.1824	0.3626	0.0842
	abcf	0.961	0.286	0.013	6.741	0.6262	0.1812	0.3684	0.0766
*x* = 5		0.959	0.291	0.013	6.737	0.6256	0.1798	0.4458	—

**Fig. 3:**
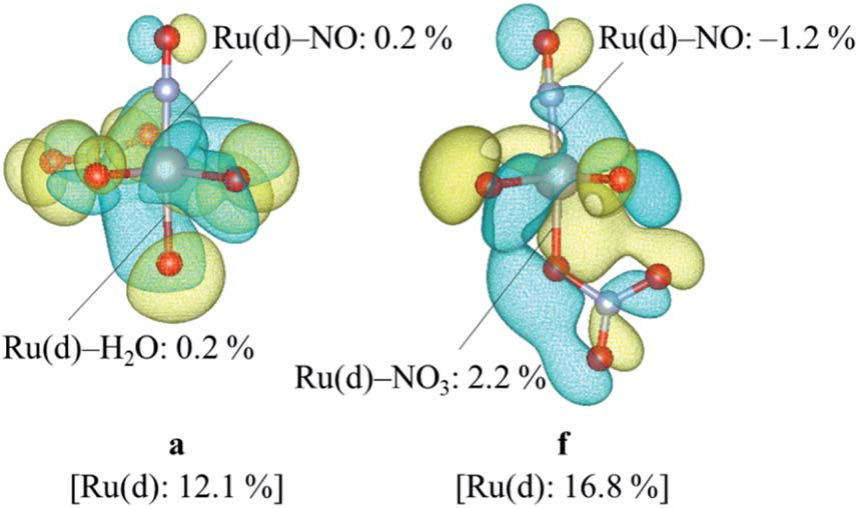
MO surface descriptions of HOMO-9 of the complexes a and f with the DOS values.

We focused on the difference in the Ru–NO bond strength between the complexes a and f. Partial density of states, PDOS, of Ru d-orbitals and bond overlap density of states, BODOS, between Ru d-orbitals and the donor atoms of the ligands were estimated by using our previous method,^21^ which was based on Mulliken population analysis,^47^ and the analytical procedure and numerical data are shown in ESI.† The Ru d-orbitals for both the complexes a and f in the valence orbitals region were distributed into the MO region from HOMO–27 (mo36) to HOMO (mo63). The sum of PDOS was *ca.* 320%, which consists of *ca.* 70% of e^b^_g_-type (d_*z*^2^_, d_*x*^2^−*y*^2^_) and *ca.* 250% of t_2g_-type (d_*xy*_, d_*yz*_, d_*zx*_) MOs. This ratio was consistent with the previous DFT result of [Ru(NO)L_5_] (L = Br^−^, Cl^−^, NH_3_).^21^ The contribution of BODOS between the Ru d-orbitals and the nitrogen atom of NO^+^ to the sum of PDOS was 13.3 and 11.9% for the complexes a and f, respectively. This indicates that the Ru–NO^+^ bond has positive bond overlap (bonding-type) interaction and the bond overlap of the complex a is larger than that of the complex f. Fig. 3 shows the MO surfaces of HOMO−9, which is one of t_2g_-type MOs, with the values of PDOS and BODOS for the complexes a and f described at 1.4 × 10^−4^ e bohr^−3^. This indicates that the BODOS values of the Ru–NO bond is negative for the complex f, indicating the antibonding interaction. This may originate to the greater bond strength of the *trans* Ru–NO_3_ bond in the complex f than that of the *trans* Ru–H_2_O bond in the complex a, indicated as the BODOS values in Fig. 3 and the *ρ*_BCP_ values, 0.105 and 0.085 e bohr^−3^ for Ru–NO_3_ and Ru–H_2_O bonds, respectively. This suggests that the larger σ-donor ability of NO_3_^−^ ligand than H_2_O leads to making the Ru(d_π_)–NO bond to be antibonding interaction, resulting in weakening the Ru–NO bond, known to be “*trans*-influence”.^50^ We suggested that the Ru–NO bond weakening destabilizes the Gibbs energy of the complex f to give only the complex a.

### Complexation kinetics between reactions a → ab and a → ac

**Fig. 4:**
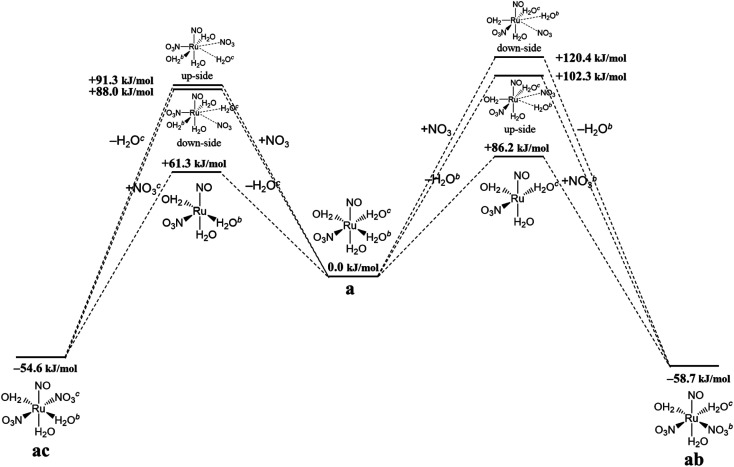
Reaction diagram in the stepwise complexation from the complex a to the complexes ab and ac.

Finally, we discuss the difference in the reaction rates of stepwise complexation reaction from the complex a to the complexes ab and ac, denoted as *k*^a→ab^ and *k*^a→ac^, respectively. Scargill *et al.* indicated experimentally that *k*^a→ab^ was slower than *k*^a→ac^, although the stability of the complex ab was higher than that of the complex ac.^12^ In case of octahedral system with d^6^ low-spin state, such as the present Ru complexes, it is well-known that the complexation reaction proceeds *via* the bond dissociation at first to give a tetragonal pyramid type transition state followed by substitution reaction, *i.e. S*_N_1 mechanism from a viewpoint of crystal field theory.^51^ This is based on that *S*_N_1 and *S*_N_2 substitution mechanisms proceed *via* transition states of square pyramid and pentagonal bipyramid, respectively, the crystal field stabilization energy of *S*_N_1 transition state is higher than that of *S*_N_2 transition state. Here we assumed the activation energy of the stepwise complexation reaction, *E*_a_, as the binding energy between the Ru and H_2_O, which is to be substituted, and compared the binding energy of the Ru–H_2_O bond between at *b* and *c* positions. Fig. 4 shows the reaction diagram for a → ab and a → ac. The activation Gibbs free energies based on binding energies were estimated as 86.2 and 61.3 kJ mol^−1^ during a → ab and a → ac reaction steps, respectively. This indicates that the stepwise complexation for a → ab proceeds with the higher reaction barrier than that for a → ac, being qualitatively consistent with the experiment.^12^ The difference in the activation free energies between a → ab and a → ac is considered to be a consequence of a classic example of *trans*-influence combined with trans effect.^52^ The result from the calculated bond length for the complex a indicated the shorter Ru–O_b,d_(H_2_O) length than the Ru–O_c_(H_2_O) shown in Table S1,† meaning the stronger *trans*-influence of NO_3_^−^ donor than H_2_O donor. We suggest that this made the Ru–O_c_(H_2_O) bond relatively weaker to give the lower activation free energy, *i.e.* “trans-effect”. In addition, we discuss the existence of hydrogen bond between NO_3_^−^ and H_2_O, both which bonded to the Ru at the *a* and *b* positions linked with HO–H⋯ONO_2_^−^, in the complex a. The O(NO_3_)⋯O(H_2_O) and O(NO_3_)⋯H(H_2_O) distances were 2.605 Å and 1.732 Å, respectively, and the O(NO_3_)⋯O(H_2_O) distance was shorter than the experimental value, 2.885(5) Å, in pure H_2_O⋯NO_3_^−^ system.^53^ The binding energy of the hydrogen bond was 35 kJ mol^−1^ estimated by using eqn (2) in ref. 54 and well-matches to 25 kJ mol^−1^, which is the difference in the activation free energies between the reactions a → ab and a → ac. This indicates that the NO_3_^−^···H_2_O hydrogen bond originates to make the activation energy of the reaction a → ab higher compared to the reaction a → ac.

We should note that the tendency of the calculated activation free energies reproduced qualitatively the experiment, however, the absolute values of the difference, 25 kJ mol^−1^, was overestimated. The corresponding experimental values were estimated to be 3.9 and 3.7 kJ mol^−1^ in 6.1 and 8.1 M HNO_3_, respectively, at 298.15 K^12^ by using eqn (12) assuming an equal frequency factor (*A*) between the reactions a → ab and a → ac. This overestimation suggests a possibility that the substitution reactions proceed *via* the intermediate scheme between the *S*_N_1 and *S*_N_2 mechanisms, producing a seven-coordinated octahedral wedge structure as the transition state.^50^ We modeled the seven-coordinated transition states in the reactions a → ab and a → ac for up-/down-side entries of nitrate ion by relaxed surface scanning approach, in which the bond lengths between the Ru and the donor atoms of leaving and entering ligands are fixed and scanned from 2.0 Å to 3.0 Å. This result indicated that the local maximum energies were obtained when the distance between the Ru and the leaving H_2_O was 2.5 Å for the up-side nitrate entry and 2.6 Å for the down-side nitrate entry as shown in Fig. S3.† The relaxed surfaces scanning details and the thermodynamic data of the transition state models are shown in ESI.†12
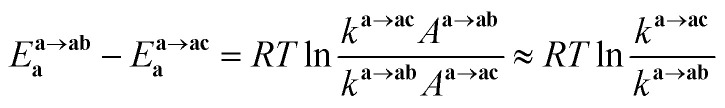


**Fig. 5:**
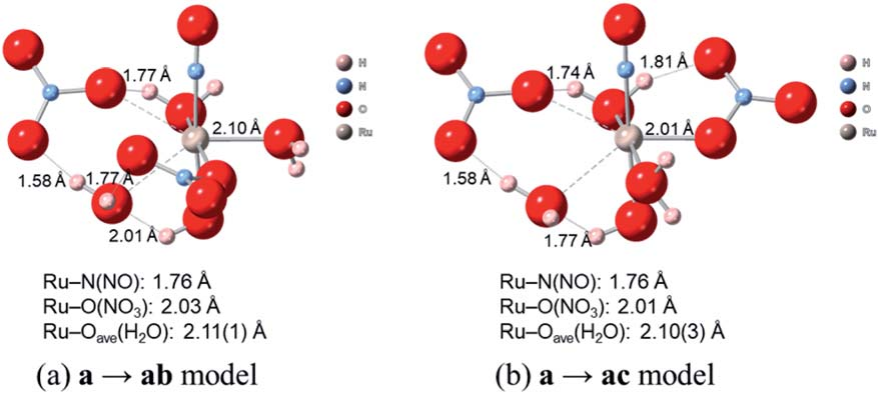
Comparison of transition state models of the reactions a → ab and a → ac.

Fig. 4 shows the activation free energies based on the seven-coordinated transition states, reproducing the experimental tendency for the both entries. This result can be also qualitatively explained by the stronger *trans*-influence combined with trans-effect of NO_3_^−^ donor than H_2_O donor as mentioned above. The difference of the activation free energies became closer, 11 kJ mol^−1^, when comparing the difference in the activation free energies of the up-side entry between the reactions a → ab (102.3 kJ mol^−1^) and a → ac (91.3 kJ mol^−1^). Their absolute values, however, were larger than those considering *S*_N_1 mechanism (Fig. 4), indicating that this substituent reaction is hard to proceed *via* this mechanism. We thought that this was caused by the entropy term of the activation free energy. The number of molecules during the reaction from the complex a to the corresponding transition state increases in the *S*_N_1 mechanism by one and decreases in this intermediate mechanism by one. This may underestimate or overestimate the activation free energies of the *S*_N_1 or this mechanism, respectively. A DFT benchmark study of thermodynamic parameters in *S*_N_2 substitution reaction at square-planar platinum(ii) complexes indicated a difficulty to reproduce the activation entropies.^55^ Actually, the activation enthalpy in the reaction a → ab was 133.8 kJ mol^−1^ for the *S*_N_1 and 56.7 kJ mol^−1^ for this intermediate mechanism, indicating the higher stability of this transition state model from a viewpoint of potential energy surface. Three-dimensional illustrations of the transition states for the up-side entry are described in Fig. 5. The coordination style of the leaving H_2_O and the entering NO_3_^−^ donated to the Ru atom has a pseudo six-membered ring linked with Ru←O–N(O)–O⋯H–O(H)→Ru for the both models as well as those for the down-side entry. When focusing on the hydrogen bonds such as NO_3_^−^···H–OH and H_2_O⋯H–OH whose lengths were shorter than sum of their van der Waals radii, 2.7 Å, the four bonds were observed in both the a → ab and a → ac models as shown in Fig. 5. The binding energies of the hydrogen bond lengths based on eqn (2) in ref. 54, as mentioned above, were 130.0 and 150.0 kJ mol^−1^ for the a → ab and a → ac models, respectively. This indicates that the stronger binding energy for the a → ac model originates in the lower activation free energy. It was also observed that the Ru–ligand bond lengths for the a → ac model were slightly shorter than those for the a → ab model, indicating another origin of the higher stability in the a → ac model. We expect that this model contributes to the computational prediction of the complexation kinetics of ruthenium complexes in nitric acid solution as well as their thermodynamics.

## Conclusions

In order to model the complexation reactions of the ruthenium-nitrosyl complexes in nitric acid solution, we have performed DFT calculations of the ruthenium-nitrosyl complexes, [Ru(NO)(NO_3_)_*x*_(H_2_O)_5−*x*_]^(3−*x*)+/−^ (*x* = 0–5), including the geometrical isomers, and reproduced the experimental observations such as EXAFS, IR spectra, and stepwise complex formation reactions in nitric acid solution. We also succeeded in understanding the relative stability and difference in reaction rate between geometrical isomers by using the bonding analyses such as bond critical point, density of states, and binding energy analyses. Finally, we demonstrated the equilibrium structures of [Ru(NO)(NO_3_)_3_(H_2_O)_2_] reproduced the experimental bond lengths and IR frequencies. Comparing the relative Gibbs energies among the geometrical isomers, the complexes with NO_3_^−^ coordinated to the equatorial positions toward Ru–NO axis were more stable than those at the axial position, being consistent with the experimental observations. The Gibbs energy difference in the stepwise complexation reaction, to which association formation energy was added, well-reproduced the fraction of the Ru species in 6 M HNO_3_ solution. Bond critical point analyses indicated that the complexes with the greater *ρ*_BCP_ value of the Ru–NO bond were more stable among the geometrical isomers. Density of states analyses suggested that the difference in the stability between the complex a and f attributed to the Ru–NO bond strength, which depended on the σ-donor ability of the ligand at the *trans* position. Furthermore, we demonstrated to estimate the activation energy in the stepwise complexation reaction a → ab and a → ac by assuming the *S*_N_1 mechanism and developed the intermediate between *S*_N_1 and *S*_N_2 mechanisms to reproduce the lower activation energy of the reaction a → ac, which was indicated to attribute to the difference in *trans*-influence combined with trans-effect between NO_3_^−^ and H_2_O donors towards ruthenium-nitrosyl ion. We expect that the present study contributes to predicting the stability and reactivity of the ruthenium-nitrosyl complexes in nitric acid solution.

## Conflicts of interest

There are no conflicts to declare.

## Supplementary Material

RA-010-D0RA05042C-s001

RA-010-D0RA05042C-s002

RA-010-D0RA05042C-s003

RA-010-D0RA05042C-s004

RA-010-D0RA05042C-s005

RA-010-D0RA05042C-s006

RA-010-D0RA05042C-s007

RA-010-D0RA05042C-s008

RA-010-D0RA05042C-s009

RA-010-D0RA05042C-s010

RA-010-D0RA05042C-s011

RA-010-D0RA05042C-s012

RA-010-D0RA05042C-s013
