# Supplementary material for: Complexation and bonding studies on [Ru(NO)(H2O)5]3+ with nitrate ions by using density functional theory calculation
Source: RSC Adv. 2020 Jun 26;10(41):24434–43. doi: 10.1039/d0ra05042c (PMC9055096; doi:10.1039/d0ra05042c)
Supplement: RA-010-D0RA05042C-s001 [file RA-010-D0RA05042C-s001.pdf]

Electronic Supplementary Material (ESI) for RSC Advances.
This journal is © The Royal Society of Chemistry 2020

Supporting Information

Complexation and bonding studies on $[Ru(NO)(H_2O)_5]^{3+}$ with nitrate ions by using density functional theory calculation

Akane Kato,[a] Masashi Kaneko[b,]* and Satoru Nakashima[a,c,]*

[a]Graduate School of Science, Hiroshima University, 1-3-1, Kagamiyama, Higashi-Hiroshima, Hiroshima 739-8526, Japan; [b]Nuclear Science and Engineering Center, Japan Atomic Energy Agency, 2-4, Shirakata, Tokai-mura, Ibaraki 319-1195, Japan; [c]Natural Science Center for Basic Research and Development, Hiroshima University, 1-4-2, Kagamiyama, Higashi-Hiroshima, Hiroshima 739-8526, Japan

*Corresponding authors: snaka@hiroshima-u.ac.jp; kaneko.masashi@jaea.go.jp

Contents:

Supporting Tables p. S2-S6
Supporting Figures p. S7-8
Supplementary Methods p. S9-S12
Gibbs energy calculation
Density of states analysis
Fitting methods of Ru fraction
Transition states searching by relaxed surface scan
References

Table S1 Calculated bond lengths (Å) and bond angles (deg.) of $[Ru(NO)(NO_3)_x(H_2O)_{5-x}]^{(3-x)+/-}$

Complexes	x = 0	x = 1		x = 2			x = 3			x = 4		x = 5
		**a**	**f**	**ab**	**ac**	**af**	**abc**	**abf**	**acf**	**abcd**	**abcf**	
Ru-NO	1.768	1.762	1.785	1.765	1.761	1.772	1.760	1.765	1.764	1.756	1.759	1.761
Ru-O_a	2.061	2.012	2.090	2.055	2.081	2.025	2.091	2.035	2.080	2.113	2.078	2.089
Ru-O_b	2.082	2.056	2.093	2.010	2.101	2.087	2.029	2.030	2.088	2.066	2.037	2.074
Ru-O_c	2.056	2.128	2.094	2.123	2.046	2.131	2.055	2.131	2.039	2.036	2.059	2.073
Ru-O_d	2.080	2.083	2.091	2.119	2.083	2.093	2.130	2.128	2.101	2.076	2.139	2.090
Ru-O_f	2.046	2.079	2.006	2.078	2.083	2.032	2.085	2.057	2.057	2.096	2.072	2.045
N-Ru-O_a	97.73	96.36	95.44	91.93	92.02	96.26	92.58	93.03	94.53	93.39	91.50	90.46
N-Ru-O_b	93.39	97.03	92.26	96.86	95.19	95.47	96.15	95.77	95.85	96.03	95.55	94.97
N-Ru-O_c	96.59	93.49	92.10	92.88	98.37	92.62	97.27	95.40	97.54	96.44	97.26	94.98
N-Ru-O_d	93.21	92.72	95.34	97.21	93.07	93.37	93.54	92.38	93.76	93.35	95.24	90.29
O_a-Ru-O_b	92.49	95.09	87.95	100.20	95.87	95.53	99.52	104.80	86.87	98.04	102.36	100.31
O_a-Ru-O_d	92.95	82.81	94.85	83.54	84.47	85.60	82.96	80.98	91.23	78.91	82.56	78.56
O_a-Ru-O_f	82.51	87.54	88.31	88.91	85.17	87.70	85.44	88.13	83.24	84.91	89.93	93.91
O_b-Ru-O_c	86.02	86.18	88.32	84.01	82.75	87.43	80.00	81.26	86.87	79.56	82.76	80.15
O_b-Ru-O_f	88.29	82.54	84.00	82.85	85.12	81.71	85.06	82.50	81.30	82.89	83.00	81.02
O_c-Ru-O_d	86.87	94.23	87.86	91.07	95.42	90.05	95.86	91.73	91.23	101.85	90.71	100.46
O_c-Ru-O_f	83.19	82.61	83.94	86.29	84.45	83.61	84.71	83.59	83.24	85.22	81.45	80.71
O_d-Ru-O_f	85.09	87.79	88.18	83.02	86.67	89.33	85.35	89.26	89.11	87.66	86.11	93.80
N-Ru-O_f	178.29	176.10	174.61	179.15	177.18	175.37	177.83	178.13	177.01	177.83	178.16	174.53
O_a-Ru-O_c	165.66	169.83	171.71	173.18	169.61	170.32	170.13	169.05	167.58	170.06	169.42	174.48
O_b-Ru-O_d	170.81	170.20	171.61	165.31	171.72	170.91	169.87	169.70	170.37	170.31	168.02	174.64
Ru-N-O	179.54	179.56	179.82	178.21	177.72	177.55	179.17	175.25	176.44	178.00	176.79	178.48

Table S2 Calculated IR frequencies and intensities of the complexes with x = 3

Vibration	**abc**		**abf**		**acf**	
	Freq. / cm^{-1}	Intensity	Freq. / cm^{-1}	Intensity	Freq. / cm^{-1}	Intensity
$\delta_{sym}(NO_3)$, Ru-H_2O rocking	737	61	737	38	712	60
	743	158	741	36	733	89
	746	152			734	169
$\nu_{sym}(NO_3)$	756	30	756	38	749	10
	767	49	762	13	752	21
	772	32	767	116	769	18
	779	46	772	133	770	18
			776	87	773	16
$\delta_{sym}(NO_3)$	883	800	889	387	892	710
	904	193	900	587	905	154
	927	546	957	486	950	396
	949	229			971	436
	977	217				
$\delta_{sym}(NO_3)$, $\nu_{sym}(NO_3)$	1236	1078	1249	902	1243	811
	1262	416	1283	278	1251	806
	1286	356	1296	730	1284	251
$\nu_{sym}(H_2O)$	1522	209	1552	154	1498	403
	1524	501	1557	138	1556	634
$\nu_{asym}(H_2O)$	1608	640	1601	649	1603	647
	1618	376	1609	879	1610	321
	1625	297	1633	219	1636	279
$\nu(NO)$	1968	1280	1954	1514	1962	1333
$\nu(H_2O)$	2948	1169	2984	1269	2826	1389
	3218	846			3114	997

Table S3 Thermodynamic data of compounds (hartree)

Compounds		E_{tot}	H_{corr}	TS	G_{corr}
x = 0		−5041.4060	0.1518	0.0525	0.0993
x = 1	**a**	−5245.5921	0.1432	0.0582	0.0850
	**f**	−5245.5869	0.1448	0.0573	0.0875
x = 2	**ab**	−5449.7678	0.1345	0.0644	0.0701
	**ac**	−5449.7685	0.1341	0.0618	0.0724
	**af**	−5449.7686	0.1353	0.0617	0.0737
x = 3	**abc**	−5653.9417	0.1260	0.0688	0.0571
	**abf**	−5653.9388	0.1265	0.0696	0.0569
	**acf**	−5653.9410	0.1262	0.0685	0.0577
x = 4	**abcd**	−5858.1035	0.1187	0.0750	0.0436
	**abcf**	−5858.1051	0.1186	0.0743	0.0444
x = 5		−6062.2608	0.1115	0.0816	0.0300
H_2O		−76.4900	0.0236	0.0215	0.0021
NO_3^-		−280.6445	0.0170	0.0286	−0.0116

Table S4 Calculated values of G_{form}, ΔG_{form}

Reaction	G_{form}(A···B) / kJ mol^{-1a}		ΔG_{form}(A···B) / kJ mol^{-1}
	Initial	Final	
x = 1 (x = 0 → **a**)	−48.1	7.0	55.1
x = 2 (**a** → **ab**)	−23.6	21.6	45.2
x = 3 (**ab** → **abc**)	3.8	46.5	42.7
x = 4 (**abc** → **abcd**)	21.5	53.9	32.4
x = 5 (**abcd** → x = 5)	13.7	43.8	30.2

Table S5 Numerical data of density of states analysis of the complexes **a** and **f**

MO number	PDOS of Ru(d) (%)		BODOS (%)			
			Ru(d)-All		Ru(d)-$N^{nitrosyl}$	
	**a**	**f**	**a**	**f**	**a**	**f**
36	7.92	8.16	3.85	4.07	3.77	3.86
37	3.18	3.31	1.58	1.54	0.02	−0.36
38	8.66	5.30	2.69	1.64	2.10	1.18
39	3.52	4.88	1.47	1.81	0.00	1.11
40	4.38	4.35	1.79	1.77	0.56	0.67
41	4.45	2.05	1.71	0.82	0.93	0.20
42	1.09	0.88	0.43	0.31	0.13	0.35
43	2.35	1.52	0.78	0.53	0.94	0.70
44	0.80	0.30	0.25	0.09	0.35	0.16
45	0.48	0.30	0.19	0.14	−0.07	−0.11
46	0.65	0.07	0.24	0.02	0.01	0.00
47	0.50	0.20	0.17	0.06	0.00	−0.02
48	17.20	9.43	5.49	2.91	0.01	−0.15
49	3.70	25.03	1.12	8.37	−1.16	−0.02
50	10.93	6.20	3.09	1.84	−0.70	−0.02
51	3.53	1.45	0.90	0.38	0.02	0.01
52	21.03	17.46	4.00	3.32	−0.10	0.27
53	18.91	9.76	3.37	1.97	−0.20	0.06
54	12.12	16.83	1.69	2.01	0.18	−1.22
55	1.64	3.82	0.17	0.22	−0.03	−0.64
56	5.98	5.47	0.62	0.52	0.11	0.02
57	19.30	18.97	1.05	1.31	0.51	0.73
58	9.34	10.45	0.51	0.50	0.14	0.14
59	6.36	0.40	−0.30	−0.02	0.46	0.03
60	45.43	50.05	−1.75	−0.85	3.01	3.08
61	36.95	32.88	−2.34	−1.77	2.00	2.69
62	17.68	2.88	-0.46	−0.59	−0.03	−0.82
63(HOMO)	51.75	79.26	−5.10	−6.87	0.37	0.02
Sum	319.81	321.66	27.21	26.05	13.34	11.94

Table S6 Stepwise complexation formation constants (K_x) for fitting models 1 and 2

$\log_{10}K_x$	Model 1	Model 2
x = 1	2.31	2.20
x = 2	1.64	1.64
x = 3	1.15	1.21
x = 4	0.94	1.00

Table S7 Thermodynamic data of transition state models (hartree)

Compounds		E_{tot}	H_{corr}	TS	G_{corr}
S_N1	**a→ab**	−5169.0452	0.1147	0.0549	0.0598
	**a→ac**	−5169.0543	0.1143	0.0549	0.0593
Intermediate up-side entry	**a→ab**	−5526.2157	0.1618	0.0694	0.0924
	**a→ac**	−5526.2199	0.1616	0.0692	0.0924
Intermediate down-side entry	**a→ab**	−5526.2106	0.1629	0.0687	0.0942
	**a→ac**	−5526.2218	0.1621	0.0691	0.0930

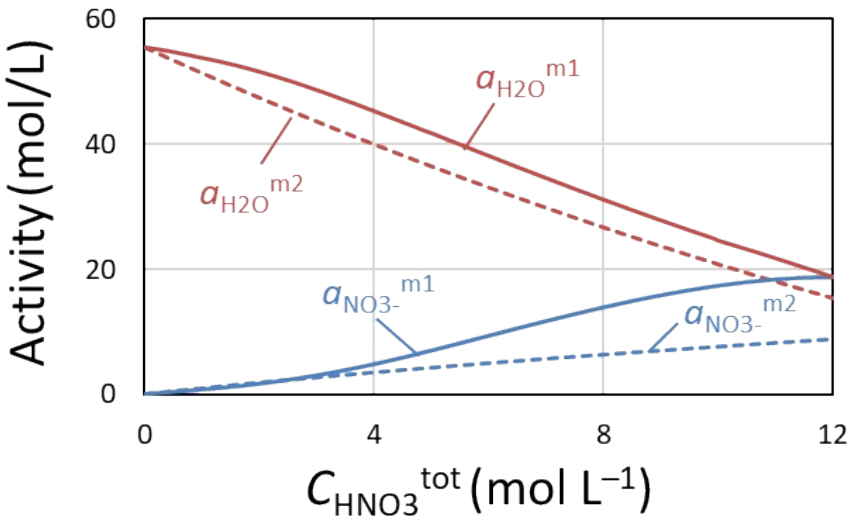

Figure S1 Dependences of a_{NO3-} and a_{H2O} on C_{HNO3}^{tot}.

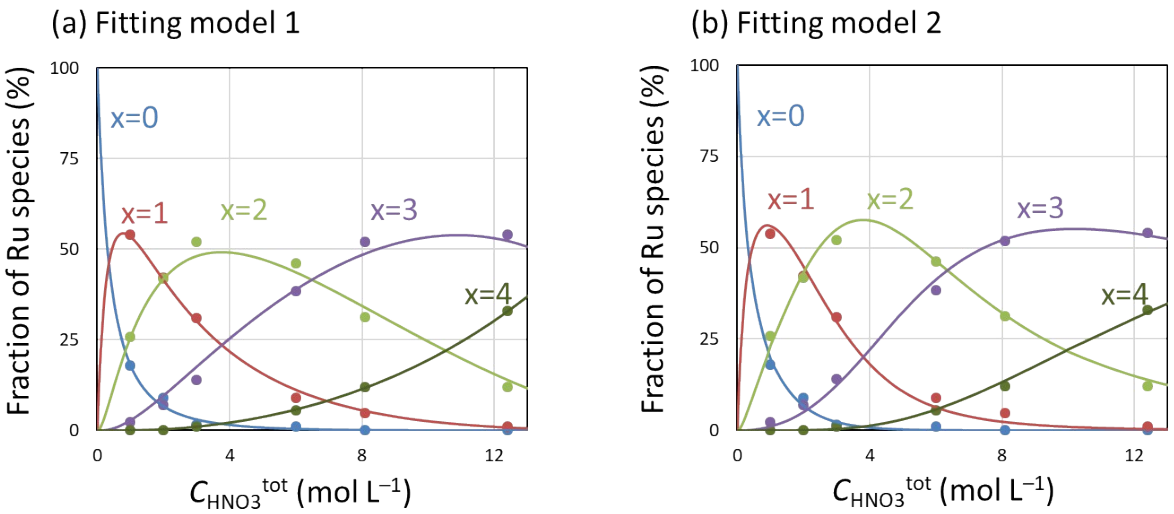

Figure S2 Fitting of speciation of Ru species on total HNO_3 concentration, in which the plot was obtained by Ref. 6.

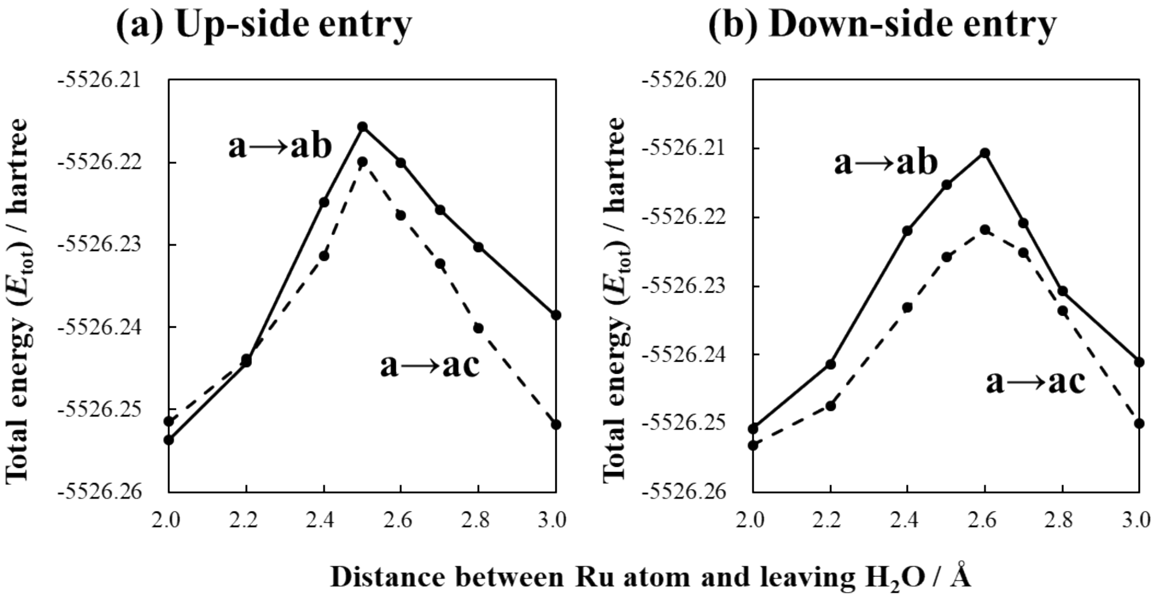

Figure S3 Transition states search in intermediate model between S_N1 and S_N2 mechanisms by relaxed surface scanning.

Supplementary Methods

Gibbs energy calculation

Standard Gibbs energy, G, can be described as sum of total energy, E_{tot}, and thermal correction to the Gibbs energy term, $G_{corr}(T)$ (eq. S1). The $G_{corr}(T)$ can be divided into thermal correction to enthalpy term, $H_{corr}(T)$, and entropy term, $S(T)$ (eq. S2). The $H_{corr}(T)$ can be divided into zero-point energy, E_{ZPE}, the contributions of vibration, $E_{vibration}(T)$, rotation, $E_{rotation}(T)$, translation, $E_{translation}(T)$, and Boltzmann thermal distribution, k_BT (k_B denotes Boltzmann constant), as shown in eq. S3. The $S(T)$ can be divided into the contributions of electron, $S_{electron}$, vibration, $S_{vibration}(T)$, $S_{rotation}(T)$, translation, $S_{translation}(T)$ (eq. S4). The contributions of vibration and rotation to enthalpy and entropy are formulated based on harmonic oscillator and rigid rotator approximations, respectively. Quasi-harmonic approximation, which was the well-known breakdown of the harmonic oscillator model for Gibbs energies of low-frequency vibrational modes, was introduced in analyzing the vibrational enthalpy and entropy terms by raising the vibrational frequencies, which are less than 60 cm^{-1} [1,2]. The derivation of formulas was referred to "Thermochemistry in *Gaussian*" by Ochterski [3].

$$G = E_{tot} + G_{corr}(T) \qquad \text{(S1)}$$
$$G_{corr}(T) = H_{corr}(T) - TS(T) \qquad \text{(S2)}$$
$$H_{corr}(T) = E_{ZPE} + E_{vibration}(T) + E_{rotation}(T) + E_{translation}(T) + k_BT \qquad \text{(S3)}$$
$$S(T) = S_{electron} + S_{vibration}(T) + S_{rotation}(T) + S_{translation}(T) \qquad \text{(S4)}$$

The E_{ZPE}, $E_{vibration}(T)$, and $S_{vibration}(T)$ in eqs. S3 and S4 are described in eqs. S5–S7, where $\Theta_v(i) = hv_i/k_B$ (h and v_i denote Planck constant and frequency of ith normal vibrational mode) denotes characteristic vibrational temperature of ith normal vibrational frequency. The $E_{rotation}(T)$ of nonlinear molecules and $E_{translation}(T)$ equal to $(3/2)k_BT$. The $S_{electron}$ is considered to correspond to spin entropy of electrons generated from spin multiplet, $2s + 1$ (s denotes spin quantum number), and equals to $k_B\{\ln(2s + 1)\}$. The $S_{rotation}(T)$ is described in eq. S8, where $\Theta_r(t)$ and σ_r denote characteristic rotational temperature of $t = x, y, z$ rotational axes and rotational symmetry number, respectively. The $S_{translation}(T)$ is described in eq. S9, where m and P denote molecular weight and pressure, respectively.

$$E_{ZPE} = k_B\Sigma_i(\Theta_{v,i}/2) \qquad \text{(S5)}$$
$$E_{vibration}(T) = k_B\Sigma_i\{\exp(\Theta_{v,i}/T) - 1\}^{-1} \qquad \text{(S6)}$$
$$S_{vibration}(T) = k_B\Sigma_i[(\Theta_v(i)/T)\{\exp(\Theta_v(i) - 1)\}^{-1} - \ln\{1 - \exp(-\Theta_v(i)/T)\}] \qquad \text{(S7)}$$
$$S_{rotation}(T) = k_B[\ln(\pi^{1/2}/\sigma_r)\{T^{3/2}(\Theta_r(x)\Theta_r(y)\Theta_r(z))^{-1/2}\} + 3/2] \qquad \text{(S8)}$$
$$S_{translation}(T) = k_B\{\ln(2\pi m k_BT/h^2)^{3/2}(k_BT/P) + 5/2\} \qquad \text{(S9)}$$

Density of states analysis

We show the analytical method of density of states (DOS) for partial DOS (PDOS) of Ru d-orbital and bond overlap DOS (BODOS) between Ru d-orbital and atomic orbitals of the donor atoms of ligands for $[Ru(NO)(NO_3)(H_2O)_4]^{2+}$ whose values were employed in **Figure 3**. This method is based on Mulliken population analysis [4]. The DOS values of the ith MO, $N(i)$, is calculated by eq. S19, where $P_{\mu\nu}$ and $S_{\mu\nu}$ denote the density matrix and the overlap matrix between basis functions ψ_μ and ψ_ν, respectively.

$$N(i) = \Sigma_\mu \Sigma_\nu P_{\mu\nu}(i) S_{\mu\nu} \qquad \text{(S19)}$$

PDOS of Ru d-orbital corresponds to values when the both ψ_μ and ψ_ν functions belong to Ru d-orbitals. BODOS corresponds to values when the ψ_μ and ψ_ν functions belong to Ru d-orbitals and atomic orbitals of the donor atoms of the ligands, respectively. The values of PDOS and BODOS are summarized in **Table S5**.

Fitting methods of Ru fraction

We show two fitting models to simulate the dependency of the Ru fraction on total HNO_3 concentration by using the calculated $\Delta G_x^{stepwise}$ and $\Delta G_x^{stepwise'}$ values for eqs. 10 and 11 in manuscript. Fitting model 1 is using the activities of H_2O and NO_3^-, denoted as a_{H2O} and a_{NO3-}, respectively, based on the experimentally reported data [5]. We estimated the a_{H2O} values by multiplying 55.39 mol L^{-1} (concentration of pure H_2O) with values of "Rational H_2O activity" in Table 4 of Ref. 5 for 0–12 mol L^{-1} of total HNO_3 concentration (C_{HNO3}^{tot}). The a_{HNO3} values were estimated by using the values of "Degree of dissociation" (α) and "Hypothetical activity coefficient" (y_h), which means activity coefficients of fully ionized nitric acid, in Table 4 of Ref. 5 for 0–12 mol L^{-2} of C_{HNO3}^{tot} to give eq. S10.

$$a_{NO3-}^{m1} = y_h C_{NO3-} = \alpha y_h C_{HNO3}^{tot} \qquad \text{(S10)}$$

For simplicity fitting model 2 is using the activities assuming the activity coefficients of H_2O and NO_3^- as 1. We limit to the solution condition that total Ru concentration is smaller than C_{HNO3}^{tot} and C_{H2O}^{tot} enough to be ignored (such as the experimental condition of Ref. 6 as well as HLLW solution) to give eqs. S11–S13. By combining eqs. S11–S13, acid dissociation constant of HNO_3 (K_a), and percentage by mass of HNO_3 in C_{HNO3}^{tot} (W_{HNO3}), we obtained the activities of NO_3^- and H_2O as eqs. S17 and S18, respectively. **Figure S1** shows the dependences of the activities of NO_3^- and H_2O on C_{HNO3}^{tot} for the two fitting models.

$C_{HNO3}^{tot} \approx C_{HNO3} + C_{NO3-}$ (S11)
$C_{H2O}^{tot} \approx C_{H2O} + C_{H3O+}$ (S12)
$C_{NO3-} \approx C_{H3O+}$ (S13)
$a_{NO3-}^{m2} \approx C_{NO3-} = (1/2)\{(K_a^2 + 4K_aC_{HNO3}^{tot})^{1/2} - K_a\}$ (S17)
$a_{H2O}^{m2} \approx C_{H2O} = (9.97/18)(100 - W_{HNO3}) - C_{NO3-}$ (S18)

Based on the fraction of $[Ru(NO)(NO_3)_x(H_2O)_{5-x}]$ (x = 1–4) for six experimental concentrations of C_{HNO3} [6], we obtained the K_x values that minimize the root mean square deviations of fraction of Ru species (%) between calculation and experiment. **Table S6** and **Figure S2** show the K_x values and the simulation based on the K_x values for fitting models 1 and 2. For the both fitting models, the calculated fractions of all the Ru species reproduced within ~2 % of RMSD values.

Transition states searching by relaxed surface scan
We modeled the transition state structures by using constrained geometrical optimization. Octahedral wedge geometries in which the distances between Ru atom and the leaving H_2O/ entering NO_3^- were fixed to 2.5 Å were created by using the equilibrium structures of the complex **a**. We considered the start geometries with up-side and down-side entries of NO_3^- ligand. Based on the octahedral wedge structures obtained by the constrained optimization, we scanned the potential surface of the distance between the Ru atom and the leaving H_2O from 2.0 Å to 3.0 Å by intervals of 0.1 Å with structural relaxation in which the sum of the distances between Ru atom and the leaving H_2O and between Ru atom and the entering NO_3^- were fixed to 5.0 Å. The structural relaxations were performed by the same method to the geometry optimization method in this study. The relaxed surface scanning based on the total energies by the single-point energy calculations are shown in **Figure S3**. The local maxima were obtained at 2.5 Å for the up-side entry and 2.6 Å for the down-side entry.

References
[1] R.F. Ribeiro, A. V. Marenich, C.J. Cramer, D.G. Truhlar, Use of Solution-Phase Vibrational Frequencies in Continuum Models for the Free Energy of Solvation, *J. Phys. Chem. B*, **115**, 14556–14562 (2011).
[2] B.W. McCann, N. De Silva, T.L. Windus, M.S. Gordon, B.A. Moyer, V.S. Bryantsev, B.P. Hay, Computer-Aided Molecular Design of Bis-phosphine Oxide Lanthanide Extractants, *Inorg. Chem.*, **55**, 5787–5803 (2016).
[3] J.W. Ochterski, Thermochemistry in Gaussian, Gaussian Inc Pittsburgh PA. 264 (2000) 1–19.

[4] R.S. Mulliken, Electronic population analysis on LCAO-MO molecular wave functions. III. Effects of hybridization on overlap and gross AO populations, *J. Chem. Phys.*, **23**, 2338–2342 (1955).
[5] W. Davis Jr., H.J. de Bruin, New activity coefficients of 0–100 per cent aqueous nitric acid. *J. Inorg. Nucl. Chem.*, **26**, 1069–1083 (1964).
[6] Scargill, D.; Lyon, C. E.; Large, N. R.; Flether, J. M. Nitratoaquo complexes of nitrosylruthenium III. *J. Inorg. Nucl. Chem.*, **27**, 161-171 (1965).
